# Astragalus Polysaccharide Promotes Doxorubicin-Induced Apoptosis by Reducing O-GlcNAcylation in Hepatocellular Carcinoma

**DOI:** 10.3390/cells12060866

**Published:** 2023-03-10

**Authors:** Mingzhe Li, Fangfang Duan, Zhiqiang Pan, Xiaomei Liu, Wenli Lu, Chao Liang, Zhaoqin Fang, Peike Peng, Dongwei Jia

**Affiliations:** 1 School of Basic Medical Sciences, Shanghai University of Traditional Chinese Medicine, Shanghai 201203, China; 2 Department of Pharmacology, School of Medicine, Sun Yat-sen University, Shenzhen 518107, China

**Keywords:** astragalus polysaccharide, O-GlcNAcylation, ER stress, apoptosis, Doxorubicin

## Abstract

The toxicity and side effects of chemotherapeutic drugs remain a crucial obstacle to the clinical treatment of hepatocellular carcinoma (HCC). Identifying combination therapy from Chinese herbs to enhance the sensitivity of tumors to chemotherapeutic drugs is of particular interest. Astragalus polysaccharide (APS), one of the natural active components in Astragalus membranaceus, has been reported to exhibit anti-tumor properties in diverse cancer cell lines. The aim of this study was to determine the effect of APS on Doxorubicin (Dox)-induced apoptosis in HCC and the underlying mechanism. The results showed that APS dose-dependently promoted Dox-induced apoptosis and enhanced endoplasmic reticulum (ER) stress. Additionally, APS decreased the mRNA level and protein stability of O-GlcNAc transferase (OGT), and increased the O-GlcNAcase (OGA) expression. Furthermore, OGT lentiviral transfection or PugNAc (OGA inhibitor) treatment reversed the ER stress and apoptosis induced by the combination of Dox and APS. A xenograft tumor mouse model confirmed that the combination of APS and Dox showed an advantage in inhibiting tumor growth in vivo. These findings suggested that APS promoted Dox-induced apoptosis in HCC cells through reducing the O-GlcNAcylation, which led to the exacerbation of ER stress and activation of apoptotic pathways.

## 1. Introduction

Primary hepatocellular carcinoma (HCC) is the sixth-most common and the third-most lethal type of malignant tumors, with a rising incidence worldwide [1]. Generally, clinical therapeutic strategies for HCC include surgery, liver transplantation, microwave ablation, transarterial chemoembolization and systemic chemotherapy [2]. Currently, chemotherapy is still the main option for treatment of HCC [3]. However, chemoresistance has developed in HCC patients, which presents a major obstacle to the long-term efficacy of chemotherapeutic treatments [3]. Doxorubicin (Dox) is an anthracyline chemotherapeutic agent that is widely used to treat solid tumors such as liver cancer [4]. Although the use of Dox has been somewhat limited by its side effects, recent efforts have mainly conquered chemoresistance and enhanced the sensitivity of tumors to chemotherapeutic drugs via chemosensitizers. Studies show that many traditional Chinese medicines (TCM) can help improve sensitivity to chemotherapeutic drugs, enhancing anti-tumor effects [5]. Therefore, the development of chemosensitizers from Chinese medicine would serve as references for the clinical treatment of HCC.

Astragalus membranaceus (*A. membranaceus*) has a long history of medicinal use in TCM. It is now commonly used in the clinic for its beneficial effects including regulation of the immune function, anti-aging and antiviral effects, radiation protection and anti-tumor effects [6,7,8]. APS is one of the most important natural active components in *A. membranaceus*, and possesses a variety of antitumor pharmacological effects, such as enhancing immunity, inhibiting proliferation, inducing apoptosis and inhibiting the transfer of tumor cells [8]. Additionally, it is reported to exert antitumor activity in solid tumors including breast cancer [9,10], lung cancer [11], gastric cancer [12] and hepatocellular carcinoma [6,13]. In combination therapy of HCC, APS enhanced the anti-cancer effects of Dox in H22 xenograft tumor mice, which might be mediated by regulating cytokine production as well as the gene and protein expression of MDR1 [13]. The mechanism of the adjuvant antitumor effect of APS has not been fully elucidated.

O-GlcNAc modification of proteins is a unique posttranslational modification. Various nuclear and cytoplasmic proteins could be modified by O-GlcNAcylation on the free hydroxyl of select serine and threonine residues [14]. The modification cycle is mediated by the enzyme O-GlcNAc transferase (OGT), which could transfer N-acetylglucosamine to protein substrates, and the enzyme O-GlcNAcase (OGA), which remove this modification from proteins [15,16,17]. O-GlcNAcylation affects a wide variety functions of proteins, including transcription, subcellular localization, protein–protein interaction and protein stability [18]. Extensive research has shown that hyper-O-GlcNAcylation occurs in most malignant tumors, such as liver cancer, and it positively relates to oncogenesis and tumor progression [19]. In addition, reducing the level of O-GlcNAcylation can prevent cancer progression [20,21]. Studies have shown that the O-GlcNAc modification is associated with endoplasmic reticulum stress (ER stress) in many types of cancer [22,23,24]. OGT clearly induced the expression of ER stress responsive proteins GRP78 and IRE1α, which were down-regulated by OGT knockdown in NAFLD HCC cell lines [25]. Other studies have found that reducing O-GlcNAcylation led to the activation of the ER stress response in various cancer cells [22,24].

ER stress occurs when proteins cannot be folded correctly and accumulate in large amounts in the endoplasmic reticulum. If the ER stress continues, the activation of stress signals and irreversible dysfunction of the ER leads to cell apoptosis [26]. Three main pathways are involved in ER stress-induced cellular apoptosis, including the CHOP, caspase-12 and IRE1-ASK1-JNK pathways [27,28,29]. It has been reported that the CHOP pathway is pivotal in endoplasmic reticulum stress-induced apoptosis in neoplastic disease [30,31]. CHOP is involved in mitochondria-dependent apoptosis, in which the protein channels of the active Bcl-2 family permit apoptotic active substances (such as cytochrome C) to be released to cytoplasm [32]. Such events result in the activation of the downstream caspase family proteins, and ultimately lead to cell apoptosis.

Herein, we demonstrated that APS enhanced Dox-induced apoptosis through decreasing the intracellular O-GlcNAcylation and inducing the ER stress response. The combination treatment of Dox and APS efficiently inhibited the growth of xenograft tumors in vivo. Our data may reveal the potential of APS as a chemotherapy sensitizer in the treatment of HCC. Moreover, the role of APS in reducing OGT expression and increasing OGA expression allows APS to play an extensive role in cancer therapy.

## 2. Materials and Methods

### 2.1. Cell Culture and Reagents for Cell Treatment

Hep3B and L02 cells were obtained from the Cell Bank of CAS (Shanghai, China). Cells were grown in DMEM, plus 10% FBS and 1% Penicillin-Streptomycin solution (Gibco). APS powder (Macklin) was dissolved in DMSO, and Doxorubicin (Dox) was obtained from Sangon and dissolved in ddH_2_O.

### 2.2. Cell Viability Detection

Cells were seeded at 5 × 10^3^ each well and the viability was detected with the reagent of CCK-8 (Beyotime). Briefly, cells were cultured for 6 h, then treated with the indicated concentration of APS and/or Dox for 24 h. CCK-8 reagent was added into the wells and incubated for 30 min, then the absorbance of each well was measured at 450 nm. Each experiment was repeated three times with four duplicated wells in each group.

### 2.3. Flow Cytometry

Cell apoptosis was analyzed using flow cytometry with an apoptosis detection kit (BD Company, Franklin Lakes, NJ, USA). Briefly, cells were stained with FITC and PI for 30 min, then resuspended with 1× binding solution. The samples were detected using a CytoFLEX flow cytometer (Beckman Coulter, Brea, CA, USA).

### 2.4. Western Blot

Proteins extracted from cells and tumor tissues were subjected to quantification and electrophoresis, then transferred to PVDF membranes. The membranes were subjected to incubating with 5% skimmed milk, specific primary antibodies and corresponding second antibodies. The primary antibodies were as follows: Rabbit anti-Cleaved Caspase-3 (#9664), -Bim (#2933), -Bax (#41162), -CHOP (#5554), -phospho-PERK (#3179), -phospho-eIF2α (#3398), -β-actin (#4967) antibodies; mouse anti-Bcl-2 (#15071) and -CTD110.6 (#9875) antibodies were purchased from CST. Rabbit antibodies of anti-OGA (ab124807), -P-gp (ab170904) and -GFPT1 (ab125069) were obtained from abcam. Antibody of OGT (11576-2-AP) was purchased from Proteintech. Mouse-anti-RL2 (MAI-072) antibody was purchased from Thermo Scientific. 

### 2.5. Lentivirus Transfection

Hep3B cells were transfected with lentivirus carrying *ogt* or vector control by HitransG A (Genechem Company, Shanghai, China) according to the manufacturer’s instructions. Briefly, cells were cultured in complete medium containing lentiviral particle stock and HitransG A for 16 h, then cultured in complete medium for another 56 h. The stably infected cells were obtained by screening with puromycin and infection efficiency was identified through Western blot analysis.

### 2.6. qRT-PCR Detection

Total RNA was extracted and reverse-transcribed to cDNA with a reverse transcriptional kit (Takara). qPCR was performed using the kit of TB Green (Takara) and a Quant Studio 3 machine. The gene expression was normalized to GAPDH level and expressed as relative values. Designed primers were synthesized by Thermo Scientific. All primer sequences are shown in Table S1.

### 2.7. Immunofluorescence

Hep3B cells were seeded in a confocal dish and received the indicated treatment for 24 h. Cells were fixed with 4% paraformaldehyde, permeabilized with 0.2% Triton X-100 for 5 min and blocked with 1% BSA for 1 h. Then, the samples were incubated with primary antibodies for 18 h at 4 °C, followed by incubating with secondary antibodies at 37 °C in the dark. Cellular nuclei were counterstained with Hoechst 33342 (Invitrogen, Waltham, MA, USA). Images were captured through laser confocal microscopy (Leica, Deer Park, IL, USA). The primary antibodies including rabbit anti-Bax (#41162) and mouse anti-CHOP (#2895) were purchased from Cell Signaling Technology, and rabbit anti-Bip (ab21685) was purchased from abcam.

### 2.8. Animal Study

Animal experiments were carried out in accordance with the approved guidelines of the research medical ethics committee of Shanghai University of Traditional Chinese Medicine, ethical approval reference number: PZSHUTCM220711026. Male BALB/c nude mice (4–6 weeks) purchased from Shanghai Slake were housed in SPF microbiological status. To establish the Hep3B xenograft tumors model, approximately 2 × 10^6^ Hep3B cells were harvested and resuspended in 100 μL saline and injected subcutaneously into the flanks of each mouse at day 0. Seven days after the inoculation, the mice were randomly divided into four groups (five mice per group): control group, APS group, Dox group and APS + Dox group. A total of 50 mg/kg APS and/or 2 mg/kg Dox were administered via intraperitoneal injection every three days until day 28. Tumor size was measured every four days, and tumor volume was calculated with the formula: V = ab^2^/2, length (a) and width (b).

### 2.9. Immunohistochemistry

The tumor tissues were embedded with paraffin and subjected to antigen retrieval in boiling citrate buffer. The slides were subjected to incubation with block solution for 5 min, primary antibodies for 18 h at 4 °C and HRP polymer for 10 min. Subsequently, the sections were treated with DAB reagent and nuclear staining with hematoxylin. The antibodies of Cleaved Caspase-3 (#9661) and CHOP (#2895) were obtained from CST, OGA (ab124807) was purchased from abcam and OGT (11576-2-AP) was purchased from Proteintech.

### 2.10. Statistical Analysis

The results are given as means ± SEM. One-way ANOVA analysis is used for statistical analysis with SPSS 22.0 software. A value of *p* < 0.05 was regarded as statistically significant.

## 3. Results

### 3.1. APS Enhances Dox-Induced Cell Death in Hepatocellular Carcinoma Hep3B Cells 

**Figure 1:**
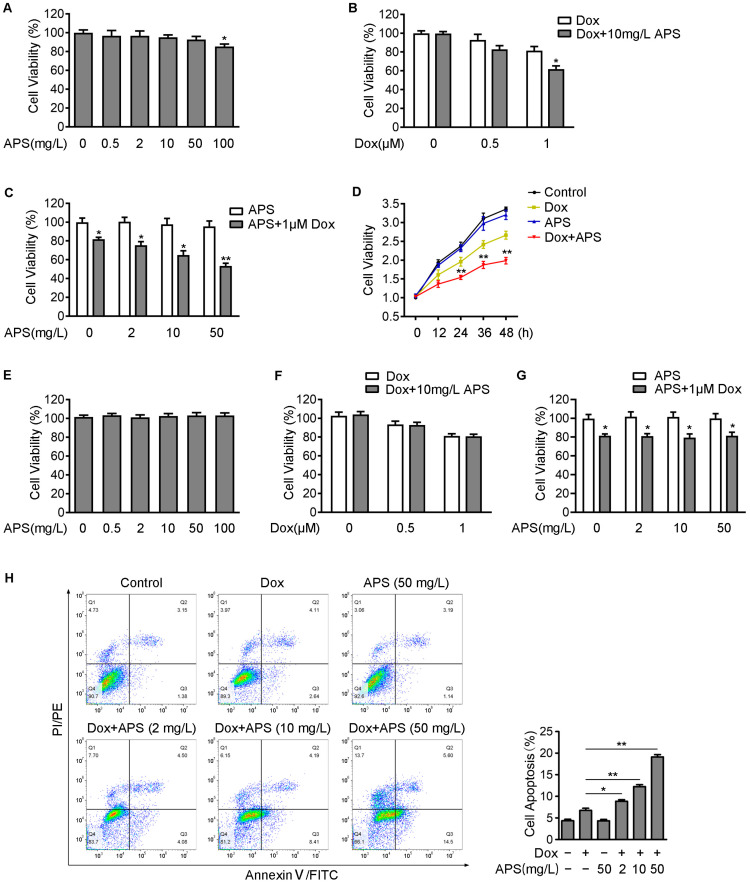
APS enhanced Dox-induced cell death in hepatocellular carcinoma Hep3B cells. Hep3B cells (**A**) or L02 cells (**E**) were treated with APS (0, 0.5, 2, 10, 50, 100 mg/L) and analyzed using a CCK-8 assay. In the presence or absence of 10 mg/L APS, Hep3B cells (**B**) or L02 cells (**F**) were treated with Dox (0, 0.5, 1 μM) for 24 h, and subjected to CCK-8 detection. Hep3B cells (**C**) and L02 cells (**G**) were treated with APS (0, 2, 10, 50 mg/L) in the absence or presence of 1 μM Dox for 24 h, and cell viability was determined using CCK-8 assay. (**D**) Hep3B cells were cultured with normal medium (control group), 1 μM Dox, 50 mg/L APS and 1 μM Dox + 50 mg/L APS for 48 h and assayed for cell viability every 12 h. (**H**) Hep3B cells were treated with 1 μM Dox along with or without increasing doses of APS (2, 10, 50 mg/L), and a flow cytometry assay was used to detect the cell apoptosis. Data are expressed as mean ± SEM from three independent experiments. *, *p* < 0.05, **, *p* < 0.01.

In this study, we first tried to determine whether APS could suppress cell viability of hepatocellular carcinoma Hep3B cells. Cells were treated with a series of concentrations of APS (0–100 mg/L). The CCK-8 assay showed that 0–50 mg/L APS had little effect on cell viability, and only 100 mg/L APS could slightly impair the viability of Hep3B cells (Figure 1A). Furthermore, we determined the viability of Hep3B cells in response to a range of Dox and APS concentrations. As shown in Figure 1B, the cell viability was only reduced to approximately 80% at 1 μM of Dox. However, the cell survival rate was suppressed to about 60% when treating with combination of 1 μM Dox and 10 mg/L APS. On the other hand, treatment with APS (0–50 mg/L) alone had little effect on cell survival, but 0–50 mg/L APS combined with 1 μM Dox dramatically decreased the cell survival dose-dependently compared to APS alone (Figure 1C). In addition, cell proliferation was also examined using the CCK-8 assay. As shown in Figure 1D, the combination of Dox and APS remarkably suppressed cell proliferation compared with the Dox group. Additionally, no significant difference was observed between the APS group and control group. To determine the role of APS on the cell viability of normal cells, we treated L02 cells with a range of concentrations of APS. The result of CCK-8 showed that L02 cell viability was not affected by 0–100 mg/L APS treatment (Figure 1E). Inconsistent with Hep3B cells, 10 mg/L APS in combination with Dox (0–1 µM) did not show synergistic inhibitory effects on L02 cell viability (Figure 1F). The combination treatment of 0–50 mg/L APS with 1 μM Dox decreased all cell viability to about 80% in L02 cells. However, no concentration dependence was observed with the combination of 2, 10 and 50 mg/L APS with 1 μM Dox in terms of L02 cell viability (Figure 1G). Next, cell apoptosis was detected using flow cytometry, and we found that percentage of apoptosis cells was increased by Dox treatment, and the combination of Dox and APS dose-dependently promoted the apoptosis compared with Dox treatment alone (Figure 1H). These data implied that the combination treatment of Dox and APS down-regulated the cell viability of Hep3B, and APS dose-dependently enhanced Dox-induced apoptosis. 

### 3.2. APS Induces ER Stress Response and Enhances the Dox-Induced Apoptosis in Hep3B Cells

**Figure 2:**
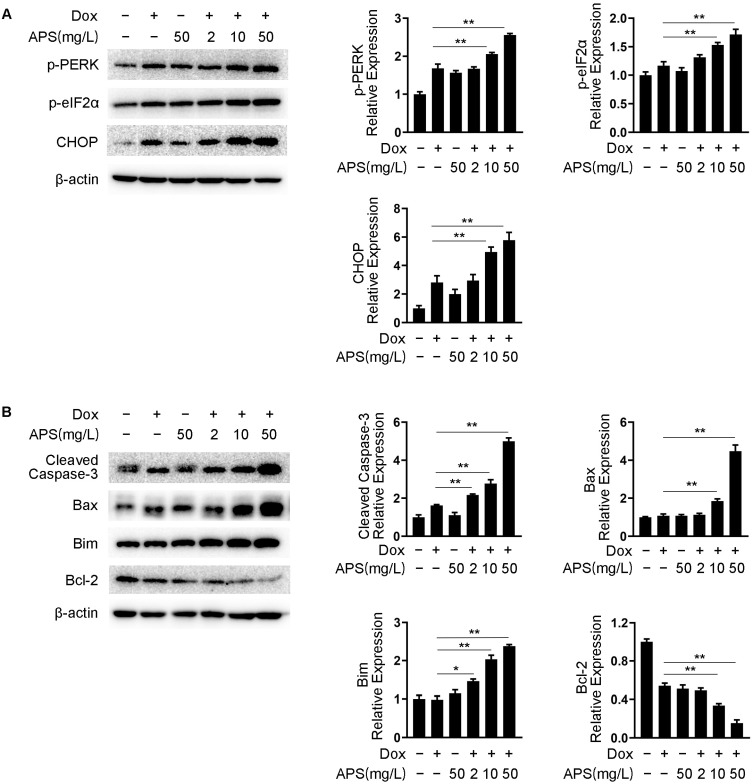
APS induces ER stress response and enhances the Dox-induced apoptosis in Hep3B cells. Hep3B cells were treated with 1 μM Dox along with or without increasing doses of APS (2, 10, 50 mg/L). (**A**) ER stress-responsive proteins were examined using Western blots. (**B**) Apoptosis-related proteins were examined using Western blots. Data are expressed as mean ± SEM from three independent experiments. *, *p* < 0.05, **, *p* < 0.01.

Studies have demonstrated that Dox induces the activation of the ER stress pathway in tumor cells, which is one of the most common mechanisms that leads to the reduction in chemotherapy sensitivity in HCC [33,34]. We first examined the effect of APS on the expression of ER stress signaling proteins in the absence or presence of Dox in Hep3B cells. As shown in Figure 2A, 50 mg/L APS slightly up-regulated the expression of p-PERK, p-eIF2α and CHOP. As expected, Dox treatment could induce the activation of the PERK pathway, and this effect was enhanced by the administration of APS in a dose-dependent manner, indicating that APS promotes ER stress signaling activation in Dox-treated Hep3B cells. The excessive and irreparable ER stress participates in the transition from survival mode to a death response, causing the activation of intrinsic apoptosis [35]. CHOP is known to promote mitochondria-mediated apoptosis by down-regulating the pro-survival protein Bcl-2 [36]. As shown in Figure 2B, treatment with 1 μM Dox decreased the level of Bcl-2 but failed to increase that of Cleaved Caspase-3, Bax and Bim. However, compared with Dox treatment alone, the combination of Dox and APS reduced the Bcl-2 level and up-regulated the level of Cleaved Caspase-3, Bax and Bim dose-dependently. Overall, these data implied that APS synergistically enhanced the activation of the ER stress response, and enhanced the Dox-induced ER stress-related apoptosis. 

### 3.3. APS Down-Regulates O-GlcNAcylation through Decreasing OGT Level and Increasing OGA Level in Hep3B Cells

**Figure 3:**
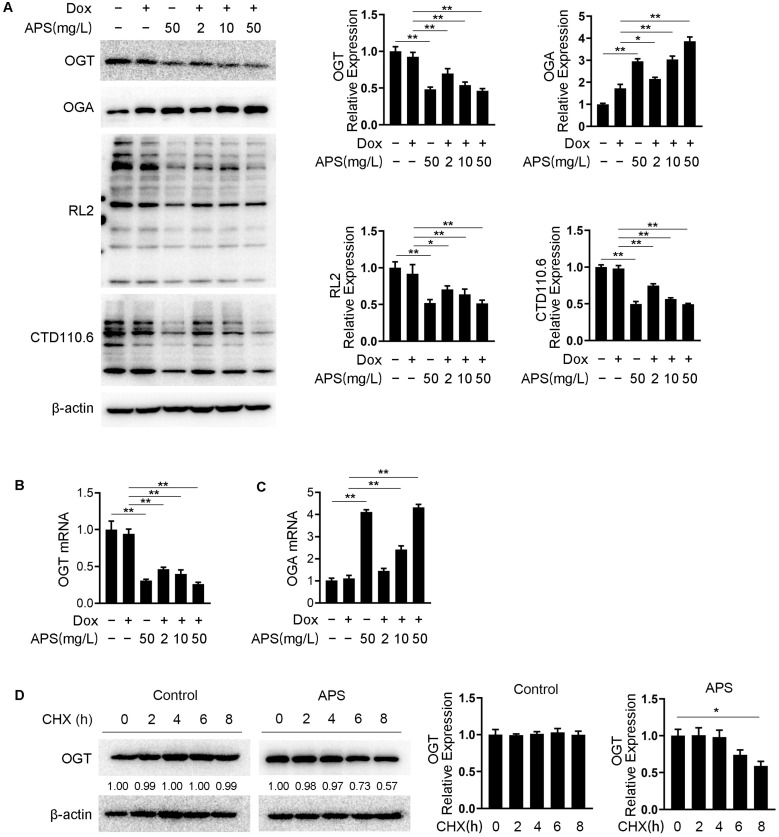
APS down-regulates O-GlcNAcylation through decreasing OGT level and increasing OGA level in Hep3B cells. Hep3B cells were treated with 1 μM Dox along with or without increasing doses of APS (2, 10, 50 mg/L). (**A**) Expression levels of OGT, OGA, RL2 and CTD110.6 were examined using Western blots. β-actin was used as an internal control. The mRNA levels of OGT (**B**) and OGA (**C**) in Hep3B cells were measured using real-time PCR. (**D**) Hep3B cells treated with or without 50 mg/L APS were subjected to Cycloheximide (CHX) chase analysis for OGT stability detection. Cells were harvested at indicated time points under 50 µM CHX treatment. Data are expressed as mean ± SEM from three independent experiments. *, *p* < 0.05, **, *p* < 0.01.

O-GlcNAcylation is reported to faciliate the survival of various types of cancer cells by regulating ER stress [37,38]. To understand the role of APS in O-GlcNAcylation in hepatocellular carcinoma cells, Hep3B cells were treated with 50 mg/L APS. As shown in Figure 3A, APS significantly elevated the protein expression of OGA and down-regulated the levels of OGT, RL2 and CTD110.6 in Hep3B cells. Compared with Dox alone, the combined use of Dox and APS decreased intracellular O-GlcNAc modification dose-dependently. Meanwhile, we found that APS treatment dramatically down-regulated the OGT mRNA level, which was also dose-dependently decreased in the combination group (Figure 3B). Additionally, the OGA mRNA levels were clearly up-regulated in both the APS and combination groups (Figure 3C). In addition, we determined whether APS could modulate the protein stability of OGT. Cycloheximide (CHX) chase analysis revealed that the OGT protein level was not influenced by CHX treatment of 8 h, which might be due to the relatively long half-life of OGT (∼12 h) [39]. Meanwhile, we observed that APS treatment clearly impaired the protein stability of OGT (Figure 3D). These data suggested that APS down-regulated the expression and protein stability of OGT, up-regulated the expression of OGA and eventually diminished the O-GlcNAcylation level of Hep3B cells. In addition, the dysregulation of HBP enzymes were related to development of cancer. HBP enzyme-targeting strategies may be an effective method for cancer treatment [40]. Therefore, we examined the effect of APS on the expression of HBP enzymes. As shown in Figure S1A, APS had no effect on the transcript expression levels of almost all HBP enzymes, including GFPT1 (the first and rate-limiting enzyme of HBP) in Hep3B cells. Similarlly, the protein levels of GFPT1 were not affected by APS or the combined use of Dox and APS (Figure S1B). 

### 3.4. APS Exacerbates ER Stress Response by Reducing O-GlcNAcylation in Hep3B Cells 

**Figure 4:**
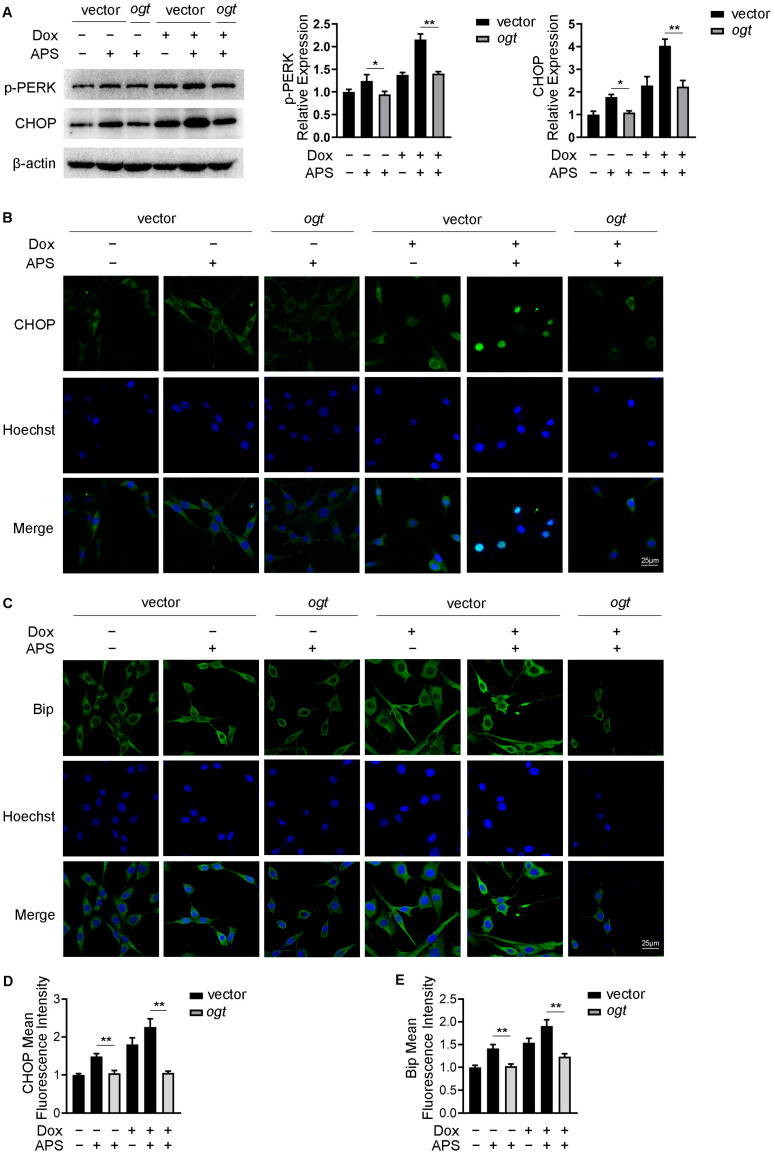
APS exacerbates ER stress response by reducing O-GlcNAcylation in Hep3B cells. Hep3B cells were transfected with lentivirius carrying vector control or ogt followed by treating with or without 50 mg/L APS, in the absence or presence of 1 μM Dox. Then, cells were subjected to (**A**) Western blot analysis for p-PERK and CHOP, and immunofluorescence for (**B**) CHOP and (**C**) Bip. Images presented are representative of each group. Mean fluorescence intensity of (**D**) CHOP and (**E**) Bip were calculated using IPP software. Scale bar, 25 μm. Five random visual fields were measured for each sample. Data are expressed as mean ± SEM from three independent experiments. *, *p* < 0.05, **, *p* < 0.01.

It has been reported that the inhibition of O-GlcNAcylation results in the enhancement of the ER stress response in tumor cells [22,24]. We next examined whether reducing O-GlcNAcylation by APS led to the activation of the ER stress response in Hep3B cells. Cells carrying lentivirius of ogt or control vector were treated with APS or in combination with Dox. Western blots showed that APS or combination treatment increased the expression of p-PERK and CHOP compared with the control group, but this effect was restored by OGT overexpression (Figure 4A). Next, an immunofluorescence assay was performed to confirm that O-GlcNAcylation is involved in ER stress regulation. As shown in Figure 4B,D, compared to the control group, the mean fluorescence intensity (MFI) and nuclear translocation of CHOP were elevated by Dox treatment, which were further strengthened in the combination group. As expected, these effects were significantly reversed by the overexpression of OGT. A similar effect was also observed in Bip staining (Figure 4C,E). Taken together, these results implied that enhancement of ER stress induced by APS alone or combination with Dox was mediated by low intracellular levels of O-GlcNAcylation. Increased levels of O-GlcNAcylation could reverse the ER stress induced by APS.

### 3.5. APS Promotes Dox-Induced Apoptosis by Decreasing Intracellular O-GlcNAc Levels

**Figure 5:**
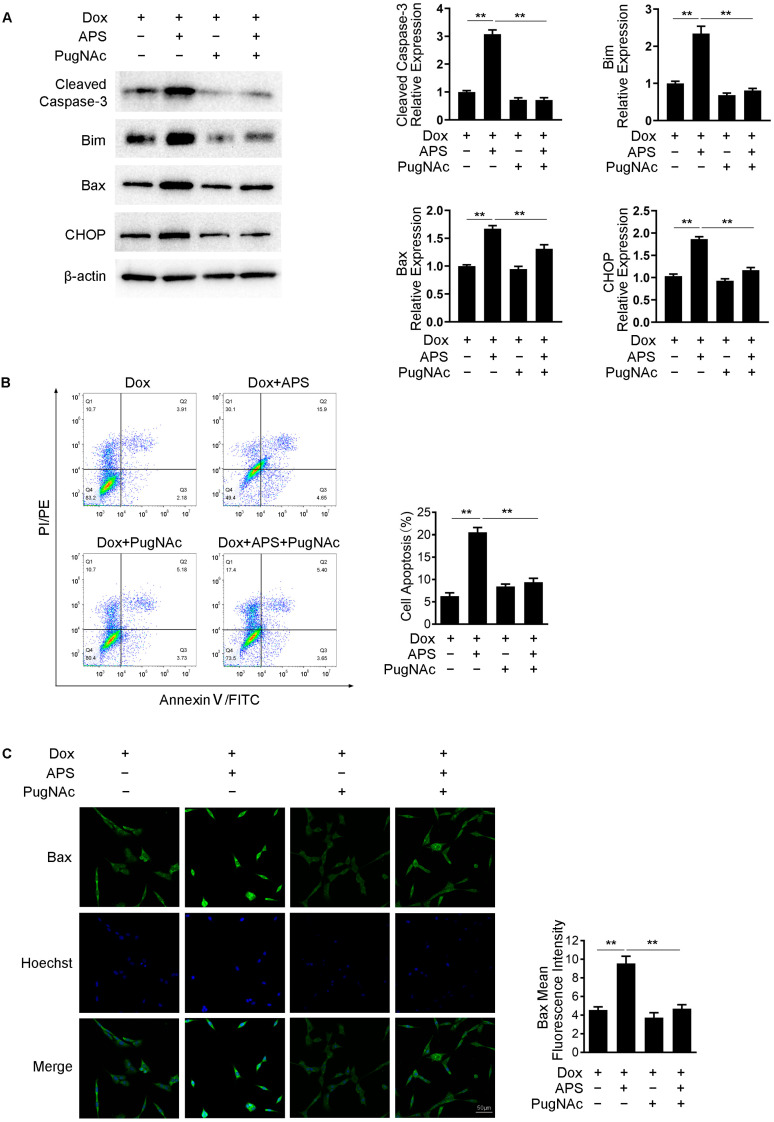
APS promotes Dox-induced apoptosis by decreasing intracellular O-GlcNAc levels. Under the treatment of 1 μM Dox, Hep3B cells were treated with or without 50 mg/L APS and in the absence or presence 10 μM PugNAc. Then, cells were subjected to (**A**) Western blot analysis for Cleaved Caspase-3, Bim, Bax and CHOP, (**B**) flow cytometry assay for cell apoptosis and (**C**) immunofluorescence for Bax. Images presented are representative of each group. Mean fluorescence intensity was measured using IPP software. Scale bar, 50 μm. Five random visual fields were measured for each sample. Data are expressed as mean ± SEM from three independent experiments. **, *p* < 0.01.

Studies have noted that decreasing the level of O-GlcNAc using inhibitors or genetic knockout of OGT would promote apoptosis in cancer cells [23,24]. We then examined whether APS could enhance apoptosis by decreasing the O-GlcNAc level. As the data mentioned above confirm, the combination of Dox and APS exhibited higher expression of Cleaved Caspase-3, Bim, Bax and CHOP in comparison with Dox treatment alone. However, this effect was attenuated by treatment with PugNAc, an OGA inhibitor, which elevated the intracellular O-GlcNAcylation (Figure 5A). The rate of cell apoptosis was detected using flow cytometry, and we found that the increased apoptosis rate in the combination group was reversed by PugNAc treatment (Figure 5B). Similar to these results, we also used immunofluorescence staining to confirm that the elevated MFI of Bax in the combination group was significantly decreased upon treatment with PugNAc (Figure 5C). These results suggested that the enhancement of apoptosis through the combination of Dox and APS was correlated with the reduction in O-GlcNAcylation caused by APS in Hep3B cells.

### 3.6. APS Strengthens the Tumor Growth Inhibitory Effect of Dox in Hep3B Xenograft Tumor

**Figure 6:**
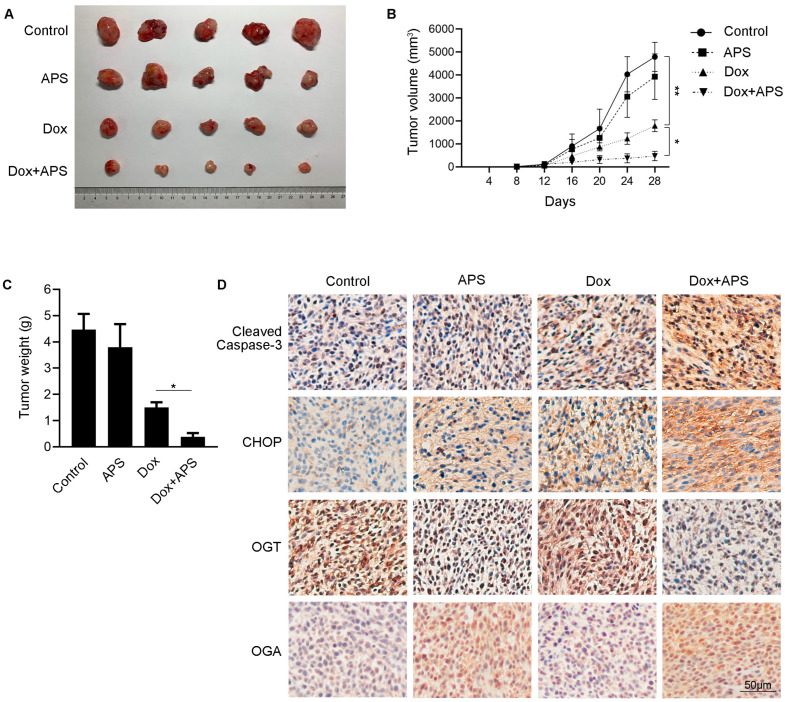
APS strengthens the tumor growth inhibitory effect of Dox in Hep3B xenograft tumor. BALB/c nude mice were inoculated with Hep3B cells subcutaneously at day 0. Mice were intraperitoneally injected at day 7; the treatment was performed every 3 days and lasted to day 28. Treatment was as follows: 1. Control (saline), 2. APS (50 mg/kg), 3. Dox (2 mg/kg), 4. Dox (2 mg/kg) + APS (50 mg/kg). (**A**) Subcutaneous tumors in BALB/c nude mice were harvested at day 28 (n = 5). (**B**) Tumor volumes (mm^3^) were monitored for 28 days by measuring with vernier caliper. (**C**) Tumor weights (g) were measured when the mice were sacrificed at day 28. (**D**) Sections of the excised tumors of each group were processed for immunohistochemistry. Scale bar, 50 μm. Data are expressed as mean ± SEM, n = 5, *, *p* < 0.05, **, *p* < 0.01.

To further confirm the combination effect of APS and Dox on liver cancer growth in vivo, a subcutaneous xenograft tumor model was established with Hep3B cells. Administration of Dox effectively reduced the tumor size and weight compared to control group, and this effect was strengthened by combining with APS. No significant inhibiting effect of tumor growth was observed in the APS group (Figure 6A–C). These data indicated that APS synergistically inhibited tumor growth with Dox in vivo. To determine the expression of Cleaved Caspase-3, CHOP, OGT and OGA in the xenograft tumor in different groups, immunohistochemistry (IHC) analysis was performed. As shown in Figure 6D, in the Dox group, the expression levels of Cleaved Caspase-3 and CHOP were higher than that of the control group. Compared with Dox administration, the expression levels of Cleaved Caspase-3 and CHOP were further enhanced. As compared with the control group, the expression of OGT was decreased and OGA was increased upon APS treatment alone or combination treatment in cancerous tissue. Similar to the in vitro results, these data indicated that APS could potentiate Dox sensitivity, and promote cell apoptosis and the ER stress response in Hep3B xenograft tumors when combined with Dox, and these effects might correlate with the down-regulation of OGT or up-regulation of OGA in tumor tissue.

## 4. Discussion

Astragalus polysaccharide (APS) is the main substance extracted from *A. membranaceus*, and it shows advantages in terms of anti-tumor effectiveness and low toxicity [41]. Studies show that APS exerts an anti-tumor role through inhibiting proliferation, inducing tumor cell apoptosis and regulating immune cell function [6,8,42]. Previous studies declared that APS was used for an adjuvant treatment to conventional chemotherapy to reduce treatment-associated adverse effects in patients [43], or to increase the tumor response to chemotherapies [13,44,45]. For instance, APS exerts a synergistic anti-tumor effect with adriamycin by enhancing the expression of cytokines or down-regulating the *MDR1* mRNA level in gastric cancer or H22-bearing mice [13,44]. It is also confirmed in this study that APS decreased the elevated expression of *MDR1* and P-glycoprotein induced by Dox in Hep3B cells (Figure S2). Nevertheless, the potential synergistical antitumor effect of APS on HCC and protein stability has not been fully elucidated. In the present study, we found that APS could enhance the apoptosis induced by Dox in hepatocellular carcinoma Hep3B cells in vivo and in vitro. More detailed studies revealed that APS exacerbated ER stress by down-regulating O-GlcNAcylation under Dox treatment, and finally promoted apoptosis in Hep3B cells. 

In cancer cells, glucose metabolism could be reprogrammed to obtain energy through glycolysis even under aerobic conditions and the activate the HBP pathway, which produces UDP-GlcNAc as the substrate for O-GlcNAc modification [46,47]. Aberrant elevated O-GlcNAcylation is related to the proliferation, progression and metastasis of cancer cells in various cancers including those of the breast, colon, pancreas, liver and lung [47,48,49]. Therefore, further research is needed to find potential therapeutic agents targeting hyper-O-GlcNAcylation [38]. Investigational OGT inhibitor is an ideal potential therapeutic option for cancers [25,50]. For example, OSMI-1, one of the OGT inhibitors, combined with Dox synergistically increased the apoptosis of HepG2 cells [23]. However, the off-target and toxic side effects of some OGT inhibitors prevent their application in vivo [51]. In this study, we found APS alone or in combination with Dox reduced the level of O-GlcNAcylation. Further study revealed that APS down-regulated the expression of OGT by decreasing the mRNA level and reducing the protein stability of OGT (Figure 3). A polysaccharide fraction from *A. membranaceus* has been confirmed to be safe through genotoxicity assays and an oral toxicity test with the NOAEL (no observed adverse effect level) of 5000 mg/kg/day for rats, which is a dose 30~40 times as high as the effective oral dose in humans [52]. Thus, APS may be used as a potential safe agent for reducing O-GlcNAc in cancer therapy in vivo. On the other hand, the biosynthesis of UDP-GlcNAc can be reduced by targeting the rate-determining enzyme of HBP, thereby starving OGT of its substrate. However, no significant inhibitory effect of APS was observed on these enzymes. We found that the reduction in O-GlcNAcylation by APS is mainly through regulating OGT and OGA.

In ER stress, the activation of ER signaling recruits chaperones to facilitate proteins’ folding capacity and degrade misfolded proteins [45]. When the accumulation of misfolded proteins cannot be prevented, the activation of CHOP or eIF2α signals ultimately leads to cell apoptosis [53]. Transcription factor CHOP is involved in the regulation of genes that are responsible for cell apoptosis [54]. A variety of anticancer agents could induce the ER stress response, which strengthened or attenuated the anticancer effect depending on tumor type or tumor environment [55]. In our study, Dox treatment induced ER stress with the activation of PERK/p-eIF2α and increased the expression of CHOP in Hep3B cells. It was observed that APS increased the ER stress response, which was dramatically enhanced by the combination treatment of Dox and APS (Figure 2). Our finding is different from a previous study, which suggested that APS effectively suppressed UPR through inhibiting the PERK-eIF2α pathway in colon cancer cells [56]. Hence, further exploration of the effect of APS on ER stress in various cancer cells is needed.

Recently, many reports have suggested that O-GlcNAcylation leads to the ER stress response [57]. In turn, various stresses (including ER stress and chemotherapy) increase the intracellular O-GlcNAcylation and directly affect the survival of cancer cells [58]. Studies have shown that a low level of O-GlcNAcylation leads to the ER stress response in both HCC and breast cancer cells [23,24]. Similarly, this study revealed that APS decreased the O-GlcNAc level and exacerbated the Dox-induced ER stress, accompanied by the up-regulation of p-PERK, p-e-IF2α, CHOP and Bip in Hep3B cells, which was dramatically recovered by OGT overexpression (Figure 2A and Figure 4). In fact, key molecules in the ER stress pathway such as eIF2α were reported to be modified by O-GlcNAc, which inhibits the phosphorylation of eIF2α and protects cells against ER stress-induced apoptosis [58]. Therefore, further studies are needed concerning whether APS is involved in regulating O-GlcNAc modification of critical molecules in ER stress.

The O-GlcNAc modification of proteins is part of a pro-survival signaling program. Conversely, reducing O-GlcNAcylation levels sensitizes cells and tissues to injury [59,60,61]. Interestingly, our study showed that 50 mg/L APS reduced the O-GlcNAc level, but had no effect on cell viability and apoptosis (Figure 1). This phenomenon may be attributed to the insufficient activation of ER stress induced by APS, which was confirmed by the slight increased expression of PERK/eIF2α/CHOP upon APS treatment as mentioned previously. These findings are inconsistent with previous studies, which have suggested that APS promotes apoptosis in a number of cancer cells [38]. In this study, 1 μM Dox exhibited a significant activation of the ER stress response, but failed to increase Cleaved Caspase-3, Bax and Bim, resulting in insufficient apoptotic cell death. The combination of Dox and APS further aggravated the ER stress, and the apoptotic protein and apoptotic cell rate were also increased significantly. These data suggest that the ER stress induced by lowering O-GlcNAcylation through APS is a further attack on cells in the Dox-induced stress state. In addition, the Dox doses in this animal experiment were referenced to humans. In humans, the Dox treatment dosage ranges from 8 to 400 mg/m^2^ [62]. The risk of irreversible cytotoxicity increases sharply once the total administered dose exceeds 550 mg/m^2^ [63], which is equivalent to 9.5 times higher than the total dose in this mice experiment. Therefore, the in vivo dosage of Dox in this study was moderate and relatively safe, and may provide more information for clinical decisions. As mentioned above, APS had synergistic anti-tumor effects with chemotherapy drugs in some cancers. Though aberrant O-GlcNAcylation is associated with growth, proliferation and metastasis in these cancer cells and some chemotherapies increase the O-GlcNAc level, there is no study on APS’ anti-tumor effect through regulating O-GlcNAc. According to this study, the synergistic anti-tumor effect of APS via O-GlcNAc regulation could be further studied in other cancers in the future.

## 5. Conclusions

In summary, the present study suggested that APS could enhance the sensitivity of hepatocellular carcinoma Hep3B cells to Dox and promote the efficiency of Dox in inhibiting xenograft tumor growth. APS reduced intracellular O-GlcNAcylation by down-regulating OGT expression and up-regulating OGA expression, which lead to an exacerbation of ER stress followed by related intrinsic apoptosis. The results of this study revealed that APS may be used as an optional sensitizing agent for chemotherapy for HCC.

## Data Availability

Not applicable.

## Supplementary Materials

The following supporting information can be downloaded at: https://www.mdpi.com/article/10.3390/cells12060866/s1, Figure S1: The effect of APS on the expression of HBP enzymes in Hep3B cells. Hep3B cells were treated with 1 μM Dox along with or without increasing doses of APS (2, 10, 50 mg/L). (A) Protein level of GFAT1 was detected by Western blot. β-actin was used as an internal control. (B) mRNA levels of HBP enzymes (GFAT1, GNPNAT1, PGM3, UAP1) were examined by real-time PCR. Data are expressed as mean ± SEM from three independent experiments. Figure S2: The effect of APS on the expression of MDR1 and P-glycoprotein (P-gp) in Hep3B cells or in xenograft tumor upon Dox treatment. Hep3B cells were treated with 1 μM Dox along with or without increasing doses of APS (2, 10, 50 mg/L). mRNA level of MDR1 was measured by real-time PCR (A) and P-gp was detected by Western blot (B). The tumor tissues were harvest from xenograft tumor mice mentioned above. mRNA level of MDR1 was measured by real-time PCR (C) and P-gp was detected by Western blot (D). β-actin was used as an internal control. Data are expressed as mean ± SEM from three independent experiments. *, *p* < 0.05, **, *p* < 0.01.; Table S1: Primers for quantitative Real-Time PCR.
