# Supplementary material for: Astragalus Polysaccharide Promotes Doxorubicin-Induced Apoptosis by Reducing O-GlcNAcylation in Hepatocellular Carcinoma
Source: Cells. 2023 Mar 10;12(6):866. doi: 10.3390/cells12060866 (PMC10047337; doi:10.3390/cells12060866)
Supplement: Supplementary file 1 [file cells-12-00866-s001.zip › cells-2027118-supplementary.pdf]

Supplementary Materials

Astragalus Polysaccharide Promotes Doxorubicin-Induced Apoptosis by Reducing O-GlcNAcylation in Hepatocellular Carcinoma

Mingzhe Li [1,†], Fangfang Duan [2,†], Zhiqiang Pan [1], Xiaomei Liu [1], Wenli Lu [1], Chao Liang [1], Zhaoqin Fang [1], Peike Peng [1,*,‡] and Dongwei Jia [1,*,‡]

[1] School of Basic Medical Sciences, Shanghai University of Traditional Chinese Medicine, Shanghai 201203, China
[2] Department of Pharmacology, School of Medicine, Sun Yat-sen University, Shenzhen 518107, China
* Correspondence: pengpeike@shutcm.edu.cn (P.P.); jiadongwei@shutcm.edu.cn (D.J.); Tel.: +86-21-51322117 (D.J.).
† These authors contributed equally to this work.
‡ These authors contributed equally to this work.

Table S1. Primers for quantitative Real-Time PCR.

	Primer sequence (5′ to 3′)
MDR1	Forward: TAATGCGACAGGAGATAGGCT<br>Reverse: CCGCCATTGACTGAAAGAACAT
OGT	Forward: ATCCTCTTTCTCATGGCTTCAG<br>Reverse: AACTCACATATCCTACACGCAG
OGA	Forward: AGCCAAATGGTGACAAGGAACTCTC<br>Reverse: GCTCCGACCAAGTATAACCACATCC
GFPT1	Forward: CGGGAAAGTCAAGATACCAGC<br>Reverse: CGTACACCAATCAACAGAGGG
GNPNAT1	Forward: TAGGCACCACTAAAAGACAGTG<br>Reverse: TGGCCACTATTTGCTTGTGA
PGM3	Forward: GAGGAACATGCCACCTGTTT<br>Reverse: CAAAGGCATCTTGTTGCAGA
UAP1	Forward: TACATTTATTTCACTGCCCAATT<br>Reverse: TTGCAGCTGGAAGCATTTGT
GAPDH	Forward: TGCACCACCAACTGCTTAGC<br>Reverse: GGCATGGACTGTGGTCATGAG

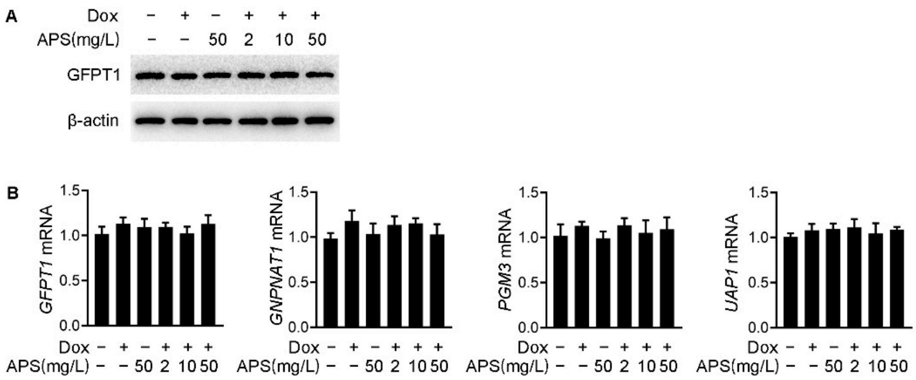

Figure S1. The effect of APS on the expression of HBP enzymes in Hep3B cells. Hep3B cells were treated with 1 μM Dox along with or without increasing doses of APS (2, 10, 50 mg/L). (A) Protein level of GFAT1 was detected by Western blot. β-actin was used as an internal control. (B) mRNA levels of HBP enzymes (GFAT1, GNPNAT1, PGM3, UAP1) were examined by real-time PCR. Data are expressed as mean ± SEM from three independent experiments.

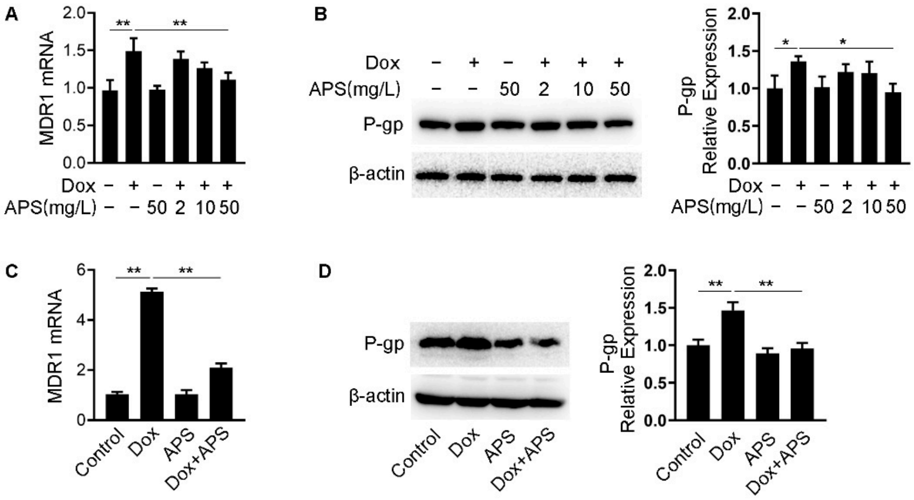

Figure S2. The effect of APS on the expression of MDR1 and P-glycoprotein (P-gp) in Hep3B cells or in xenograft tumor upon Dox treatment. Hep3B cells were treated with 1 μM Dox along with or without increasing doses of APS (2, 10, 50 mg/L). mRNA level of MDR1 was measured by real-time PCR (A) and P-gp was detected by Western blot (B). The tumor tissues were harvest from xenograft tumor mice mentioned above. mRNA level of MDR1 was measured by real-time PCR (C) and P-gp was detected by Western blot (D). β-actin was used as an internal control. Data are expressed as mean ± SEM from three independent experiments. *, $p<0.05$, **, $p<0.01$.
